# Postgraduate pharmacist development- an evaluation of Jordanian pharmacist experiences to inform and shape an evidence-based professional development policy

**DOI:** 10.1371/journal.pone.0255026

**Published:** 2021-07-27

**Authors:** Mohanad Odeh, Enas Alkhader, Alice McCloskey, Rabia Aljabra, Mohammad Al-sharayri, Faisal Al-Noimi, Majid Alarmooti, Mutazbellah Alzu’bi

**Affiliations:** 1 Department of Clinical Pharmacy and Pharmacy Practice, Pharmacy Management and Pharmaceutical Care Innovation Centre, Pharmacy School, Hashemite University, Zarqa, Jordan; 2 Pharmacy School, Middle East University, Amman, Jordan; 3 School of Pharmacy and Biomolecular Sciences, Liverpool John Moores University, Liverpool, United Kingdom; 4 Royal Medical Services, Amman, Jordan; International Medical University, MALAYSIA

## Abstract

Building capacity for developing skills as leadership, teamwork, and continuous academic support has become essential for fulfilling a successful pharmacy career. This study aims to assess Jordanian pharmacists’ views on professional development, namely: the continuous education infrastructure, strategies and programs for personal development, leadership skills, incentive schemes, drug information resources and digital services. As well as exploring options for better academic support delivered to pharmacists. To capture participant’s views, an online validated and reliable survey was developed. Non-probability sampling design was used. Participants were qualified pharmacists working at Royal Medical Services (RMS) and Community Pharmacists (CP). Comparison and descriptive statistics were used to report the results. A total of 271 pharmacists participated, 144 from RMS (8% more than the needed sample) and 127 CP (7% more than the needed sample). There was a strong desire amongst both RMS and PC groups for continuous educational training particularly in the following areas, first: Advanced counselling and communication skills (means = 8.99±0.145, CI 95% = 3.70–4.28 and 9.37±0.096, CI 95% = 4.18–4.56). Second: Personal development skills (mean = 8.92±0.142, CI 95% = 3.64–4.20 and 9.02±0.145, CI 95% = 3.73–4.30). Third, Pharmaceutical health promotion (mean = 8.05±0.180, CI 95% = 2.70–3.41 and 8.57±0.159, CI 95% = 3.26–3.89). Only 19.4% and 18.1% of the RMS and CPs respectively reported the presence of a written policy for personal development and leadership in their workplace. There were few incentives for pharmacists to participate in research. Few pharmacists used the available drug information and toxicology centers. The professional and continuous personal development of pharmacists support an evolving healthcare system. This study emphasizes the need for a tailored and documented postgraduate educational strategy, personal development, and leadership skills training in Jordan. Implementing a well-defined scheme of incentives should be encouraged to engage pharmacists in continuous professional development programs and pharmaceutical research. Such strategy and training should enhance both professional and personal performance.

## Introduction

Healthcare professionals are expected by their regulatory bodies and key stakeholders including the public, to provide patient-oriented and evidence-based medical interventions and advice. It is imperative that they remain competent in their field of practice and committed to excellence in healthcare throughout their career. Keeping their theoretical knowledge and practical skills current is essential to meet the challenges and demands of evolving societal and individual healthcare needs, health service delivery and professional responsibilities. In addition to retaining and developing theory and practice, there is increasing emphasis on personal development of the individual healthcare professional. This is achieved through enhancing transferable or so-called ‘soft’ skills e.g. improving interpersonal communication, reflective practice, problem solving and team work [1].

Pharmacists are integral members of the healthcare workforce. The role of the pharmacist however is rapidly evolving, and the boundaries between many non-medical professional and medical professional responsibilities are blurring. Previously, pharmacists were described as the ‘medicines experts’ and their role reflected this, primarily focusing around medicine procurement and supply. However, this is changing and pharmacists are increasingly patient facing and directly involved in clinical consultations and medicines management processes. Pharmacists working in hospital, community and General Practice settings should engage with patients, their careers, and other healthcare professionals to improve patient health outcomes by looking at the patient from a more holistic view including disease state, medication management, and social health and well-being. This process of direct engagement falls under the remit of the medicines reconciliation process and has shown to improve medication adherence and attitudes towards pharmacological and non-pharmacological interventions [2]. Through working collaboratively with other healthcare professionals, patients and their families a patient-centred plan is developed. Optimal pharmaceutical care has a positive impact on patients’ clinical, humanistic, and economic outcomes [3].

To achieve optimal and current pharmaceutical care pharmacists, like many healthcare professionals, are expected to engage in continuous education (CE) or continuous professional development (CPD) post-qualification. In other words, pharmaceutical training and education for pharmacists are required to maintain and improve their knowledge and meet both evolving individual patient and society’s healthcare needs [4]. In countries, such as the United Kingdom (UK) and United States (US) this has been a requirement of the professional regulators for many years. How pharmacists demonstrate their competence and continued learning is often left to the individual to decide. Unless they engage in further formal education, training resources and sessions may be limited following qualification [4, 5]. Often the terms CE and CPD are used interchangeably. CPD however is considered a more involved process of self-directed learning and perhaps more beneficial in terms of healthcare as the focus is on supporting competence through reflection and application of learning to practice. The primary goal of CE on their other hand is participation. CPD encompasses the higher levels of Blooms taxonomy of learning and encourages the cycle of reflection, planning and undertaking of learning, and evaluation of its impact on practice [6],

In Jordan, Saudi Arabia and Kuwait pharmacists complete a Bachelor of pharmacy (BPharm) degree programme [7]. Further formal pharmacy education is obtained through completion of a master’s degree program, preregistration training, a final registration examination, and -to some extent- an internship in a practice setting [8]. A recent systematic review carried out by Micallef and Kayyali [6], indicated that pharmacists prefer face to face and interactive e-learning as methods for delivery of CPD. Although there are elements of e-learning incorporated into Jordanian BPharm programs and this approach is proving useful for CPD in the UK, within Jordan there remains a major gap between formal university education and formal CPD training for those in professional practice [9]. The questions remain as to, who should provide CPD training? Should training be directly linked to area of practice only? and which delivery format is best for Jordanian pharmacists? CPD programmes across the globe tend to focus on improving knowledge around specific topics such as smoking cessation [10] herbal medicines and antibiotics [5, 11], partnership training methodologies with community partners [12], using pseudo-patients and performance feedback techniques [13]. Alternative events that may be considered as CPD include participation in workshops and conferences [14], and development of ‘soft’ skills e.g. leadership training, working with others and personal development.

Leadership has been widely discussed and identified based on several perspectives. Generally defined, leadership is a combination of position, attitude, skills, responsibilities and behaviours that enable an individual or group of individuals to “make things better” in a sustainable manner [15, 16]. Pharmacy, like other healthcare disciplines, requires appropriate leadership in order to facilitate sustainable and positive changes in pharmacy, from product-based to patient-oriented practices [17, 18].

Leadership in clinical pharmacy practice entails optimizing pharmaceutical care delivery, development of feasible remuneration models, implementation and effective activation of patient-oriented practices, professional advancement, and the strategic planning and embedding of the aforementioned principles in pharmaceutical education programs [19]. Such leadership will enable transformation of the pharmacy profession and in turn influence the societies and patients with whom pharmacists engage with [20]. Although leadership is a desirable trait for pharmacists to have, how we define leadership, teach and assess it remains inconclusive [21]

Several countries including the US and Canada have raised concerns regarding an impending potential pharmacy leadership crisis. This is attributable to the continuous evolution of practice and negligence of teaching leadership skills in pharmacy education programs. This combined with an ageing existing pharmacy leadership, leaves a gap in direction and vision for the profession [18, 22–25]. Pharmacists in many cases lost their influencing and leadership roles due to either working in small independent pharmacies where their voices are considered too small to notice, or larger chain pharmacies where such practices are limited to senior managers only rather than individuals on the frontline.

It is generally accepted that leadership skills are obtained and learned rather than inherited. There is increasing drive to improve leadership training at both undergraduate and postgraduate professional levels. In Jordan, universities follow The Accreditation Council for Pharmacy Education (ACPE). ACPE guideline 17.3 suggests not only the inclusion of leadership skills in the curriculum but also the evaluation of leadership skills for admission purposes [25]. The increasing demands are to prepare individuals that can lead rather than blindly do their professional tasks for enhancing patient centralized pharmaceutical care [26]. This encompasses employment of the required skills including; effective connections with other health practitioners and administrators, students teaching, fellows teaching, and patient pharmaceutical and clinical care [19]. To plan, explore, and assess programs, pharmacy leadership training has been discussed by researchers, where they identified a lack of original work, valid instruments for assessment, studies based on theoretical constructs, individual leadership traits and perceptions rather than demonstrations of practice over time [21]. Thus this is an area within pharmacy education where there is scope for improvement and development of appropriate training.

Working with others is crucial for success regardless of role or duties. Staff motivation, Emotional intelligence (EI), and satisfaction are major influencers of job satisfaction levels [27]. High job satisfaction and motivation are associated with increased productivity, innovation, and enhancement of an organization’s overall performance [28, 29]. Generally speaking, job satisfaction is a combination of psychological, environmental, and physiological circumstances that have a positive influence on the employee’s feelings towards his/her job [27, 30]. Various factors play a role in job satisfaction including salary, benefits, regulations, job security, work conditions, and recognition of superior performance [31]. The latter is a key factor in employees’ motivation to strive for better individual performance and outstanding work.

As pharmacist roles are expanding, the drive for further education to advance in their professional skills is aided through participation in CPD including adoption of patient counseling and clinical recommendations based on the latest evidence “Pharmacy Practice Based Research (PPBR)” [32, 33]. Pharmaceutical care and indeed healthcare and its associated research throughout the world is increasingly answerable to all stakeholders involved. It therefore must demonstrate that it is patient-centred and evidenced-based. Adopting this approach improves the quality of pharmaceutical care and contributes to widening the scope of pharmacy practice [34].

The majority of pharmacy-related literature is obtained from researches that emerged from universities, hospitals, academically affiliated clinics, pharmaceutical companies, and organizational bodies [35]. However, a shy body of literature has emerged from community pharmacies, this can be attributed to several factors including the lack of grants for community pharmacies research, lack of information regarding the willingness of their participation in research, the majority of community pharmacies are privately owned business which is, in turn, profit-oriented, lack of time, lack of support, unawareness of the available opportunities, among others [34–37].

The most recent efforts for CPD in pharmacy has been endorsed by the International Pharmaceutical Federation (FIP), which outline that it is “the responsibility of an individual pharmacist for systematic maintenance, development and broadening of knowledge, skills and attitudes to ensure continuing competence as a professional throughout their career” (Adepu and Shariff, 2010). The FIP announced the Pharmaceutical Workforce Development Goals (PWDGs), a set of 13 measurable, feasible, and achievable goals, divided into three clusters: Academy, Professional Development, and Systems, in order to facilitate pharmacy workforce development [38].

In the Middle East in general, and Jordan in particular, professional development, job satisfaction, motivation, leadership, CE, and the emerging role of pharmacists in pharmaceutical care and patient centralized management are considerably behind our European, American and Australian colleagues in particular [39]. To address this, several interventions have been made. The Pharmacists in Royal Medical Services (RMS) now choose a defined career pathway either within procurement or clinical services. Community pharmacists (CP) have increased opportunity to attend workshops sponsored or coordinated by the Jordanian Pharmacist Association (JPA) to facilitate CPD and CE. Recently the JPA announced regulations to adopt credit hours of CPD in the annual membership renewal conditions, and announce a partnership with the International Pharmaceutical Federation (FIP) to create and support the process of CPD. Subsequently, adopting similar approaches in pharmacy schools and post-graduate training from across the globe.

Considering recent changes within Jordan, this study aims to investigate and assess Jordanian pharmacists’ views about professional development including: The infrastructure for continuous pharmaceutical education and training within Jordan, Personal development and leadership, Incentives and rewards, Digital services and software for pharmacy management, Academic support and sources of information.

## Materials and methods

### Survey development

The survey was comprised of mixed mood questions type: closed ended questions, scoring questions and open ended questions (S1 Data) and designed to capture participant demographics and divided into two main sections:

**Section 1:** assessed professional development including CE infrastructure, strategies and programs for personal development and leadership skills, incentive schemes, drug information resources and digital services.

**Section 2:** investigated suggestions to enhance personal development academic support, including academic courses.

The survey was assessed for Face validation and Criterion validity [40] by the steering committee, who have experience and expertise in pharmacy practice (5 consultant pharmacists in RMS, 3 senior pharmacists that work for the JPA, and specialists in pharmacy practice in addition to authors of this paper.

Reliability measures for internal consistency and stability over time were confirmed by Cronbach’s alpha analysis and Pearson correlation respectively. The questionnaire was tested on a pilot sample of 23 responder. Responders were contacted first time and 15 days later to complete the questionnaire again (pre-/posttest reliability was performed) to justify moving forward with a large-scale pilot test. The coefficient alpha was high (Cronbach’s alpha = 0.74) [41] and very strong test-retest reliability (Pearson’s r = 0.92) [42, 43].

### Sample selection

To collect high quality and reliable data the novel non-probability sampling design "voluntary sampling design was adopted [44]. The steering committee agreed on the following target population. Inclusion criteria:

- Qualified pharmacists working at RMS (population *N* = 200)
- Qualified pharmacists working at community pharmacies and listed as Hashemite university partners for training and development (population *N* = 170)

Exclusion criteria: Pharmacists not working at RMS nor community pharmacies were excluded.

### Sample size calculation

In terms of estimating expected response numbers the Yamane Eq 1 was applied (Yamane, 1967). With a suggested E (sampling error) of 5%, the n (sample size) would be 133 for RMS group and 119 for CP group (95% confidence level and P = 0.5).


$$n=\frac{N}{1+{N(E}^{2})}$$ Eq 1

### Ethical approval and survey distribution

Ethical approval was sought for the study and granted by the Institutional Review Board of the Royal Medical Services Reference 9/24/10/2019. To capture the desired data a purposeful online survey was circulated through the Google survey platform.

Participants that met the aforementioned inclusion criteria were invited to complete the online survey by a link sent to their WHATSAPP accounts. Data collected from RMS staff between Oct-Dec 2018, and Community pharmacies June-July 2018. Within the first section of the survey participants were informed before participation that completion of the questionnaire was voluntary, they may withdraw at any time, they did not have to answer any question that they did not feel comfortable answering. It was confirmed that all submissions would be anonymous and once surveys were submitted data could not be attributed to them as an individual.

### Data analysis

Descriptive statistics (frequencies, counts, standard error of count, percentages with related standard error, Means with related standard error and 95% Confidence Intervals) were generated using SPSS software version 25 (IBM Corporation, Armonk, NY, USA). to reach quantitative conclusions. Chi square statistical tests was used to determine the association between variables where appropriate. Two-tailed t-test analysis within subjects was conducted to determine the significance of each quality attribute measured. The significance level was set at P<0.05. Free text answers were collated and thematically analyzed to reach qualitative conclusions.

## Results

A total of 271 pharmacists participated in completing the survey: 144 pharmacists who worked in RMS (8% more than the needed sample) and 127 CP (7% more than the needed sample). In both cases the expected participant response rate was surpassed.

### Participant’s demographic

The majority of participants were female: 70.1% (N = 101) for the RMS pharmacists and 72.4% (N = 92) CP. Participant age distribution is shown (Table 1)

**Table 1:** Age distribution for RMS and CP participants.

Royal Medical Services (RMS)			Community Pharmacists (CP)		
Age range	Percent (%)	N	Age Range	Percent (%)	N
22–30	30.6	44	22–30	58.3	74
31–40	44.4	64	31–40	20.5	26
41–50	22.9	33	41–50	12.6	16
51–60	2.1	3	51–60	5.5	7
61–65	0	0	61–65	0.8	1
>65	0	0	>65	1.6	2

### Specialty level and specialty fields at RMS

Pharmacists at RMS were categorized based on their specialty fields and ranking level. Unfortunately, CP in Jordan do not have this system. As seen (Table 2) most RMS pharmacists were ranked as specialists (41.7%), and there were a few participants who still under RMS training (2.8%). Regarding specialty fields, 57.6% (N = 83) of RMS pharmacists have supply related roles, and 40.3% (N = 58) have clinical roles. Three RMS pharmacists were reported as having no specialty field as they perform a mix of duties.

**Table 2:** Distribution of RMS pharmacists based on their specialty fields and specialty levels.

Specialty level			Specialty fields		
Category	Percent (%)	N	Category	Percent (%)	N
Specialist assistance	7.6	11	Clinical related	40.3	58
Senior specialist	10.4	15			
Specialist	41.7	60			
Qualify resident	7.6	11	Supply related	57.6	83
Resident	26.4	38			
Consultant	3.5	5			
Under training	2.8	4			

Ranking level to the specialty field was illustrated (Table 3). Almost similar distribution (X^2^ = 0.1577, df = 1, p = 0.69) of specialists in both fields; 25 (17.4%) and 33 (22.9%), for clinical related and supply related fields, respectively. On the other hand, the lowest proportion of clinical related employees was resident 1 (0.7%) compared to under-training pharmacists 2 (1.4%) for the supply field.

**Table 3:** Distribution of the specialty level based on the specialty fields.

	Specialty Field							
	Clinical Related				Supply Related			
Specialty Level	Count	Standard Error of Count	Percent(%)	SE (%)	Count	SE	Percent (%)	SE (%)
Specialist assistance	5	2	3.5	1.5	6	2	4.2	1.7
Senior specialist	6	2	4.2	1.7	9	3	6.3	2.0
Specialist	25	5	17.4	3.2	33	5	22.9	3.5
Qualify resident	14	4	9.7	2.5	23	4	16.0	3.1
Resident	1	1	0.7	0.7	4	2	2.8	1.4
Consultant	5	2	3.5	1.5	6	2	4.2	1.7
Under training	2	1	1.4	1.0	2	1	1.4	1.0

### Pharmaceutical education and training infrastructure

**Fig 1:**
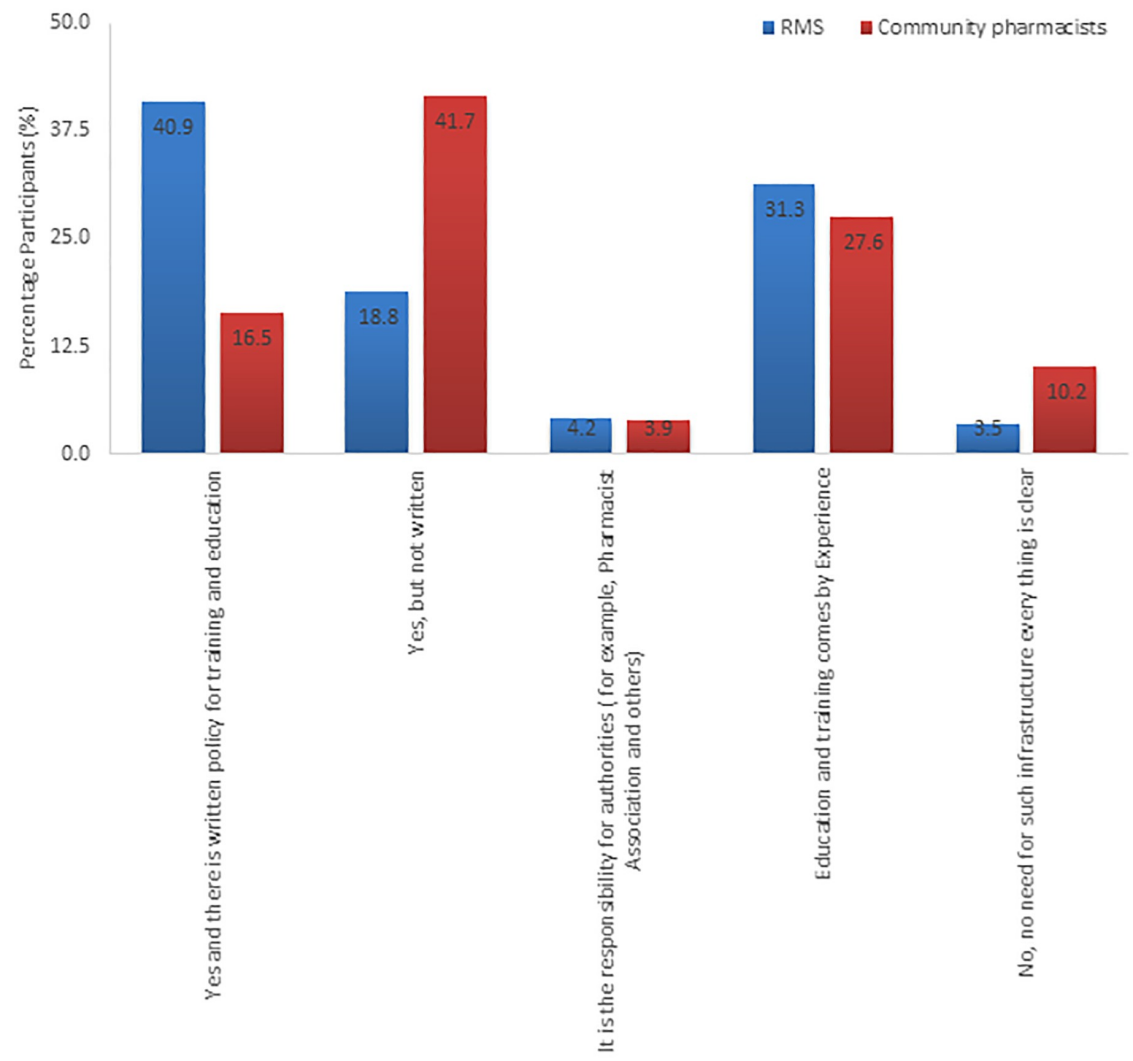
Availability of documented pharmaceutical education and training infrastructure in Royal Medical Services and Community Pharmacies.

Results showed that training was documented in a written policy for most RMS staff (40.9%) and not documented in writing for almost an equivalent number of CP (41.7%). Surprisingly, more than 55% of pharmacists had neither policy nor infrastructure for CPD and training. The proportion of CP who had a written policy was as low as 16.5% (Fig 1). Furthermore, some participants believed that pharmaceutical education and training infrastructure should be provided by authorities, 4.2% and 3.9% for RMS and CP, respectively. Written policies for pharmaceutical education and training infrastructure was significantly higher in RMS compared to CP (X^2^ = 34.811, df = 1, p<0.005).

Further analysis was carried out to compare the results within the RMS group based on their specialty field (Table 4). There was a higher percentage of a written policy for pharmaceutical education and training in supply related fields compared to the clinically related field (64.4% and 32.2%, respectively). However, 63% of RMS clinical pharmacists received non-documented pharmaceutical education and training compared to 33.3% of those in the supply field. In the same context, 39.6% (N = 57) pharmacists in RMS confirmed that the pharmaceutical education and training policies were updated, 18.1% (N = 26) denied any updates, and 19.4% (N = 28) do not know if there were any updates of the policies. There were no differences in the written policies among clinical and supply related fields within RMS pharmacists (X^2^ = 1.213, df = 1, p = 0.271)

**Table 4:** The availability of pharmaceutical education and training based on the specialty field within RMS group.

	Clinical Related				Supply Related			
Education and training at workplace	Count	Standard Error of Count	Column N %	Standard Error of Column N %	Count	Standard Error of Count	Column N %	Standard Error of Column N %
Yes and there is a written policy for training and education	19	4	32.20%	6.10%	38	5	64.40%	6.20%
Yes, but not written	17	4	63.00%	9.30%	9	3	33.30%	9.10%
It is the responsibility for authorities (for example, Pharmacist Association and others)	0	.	0.00%	.	6	2	100.00%	.
Education and training comes by experience	20	4	44.40%	7.40%	25	5	55.60%	7.40%
No, no need for such infrastructure everything is clear	2	1	40.00%	21.90%	3	2	60.00%	21.90%

### Strategies and programs for personal development and leadership skills

**Fig 2:**
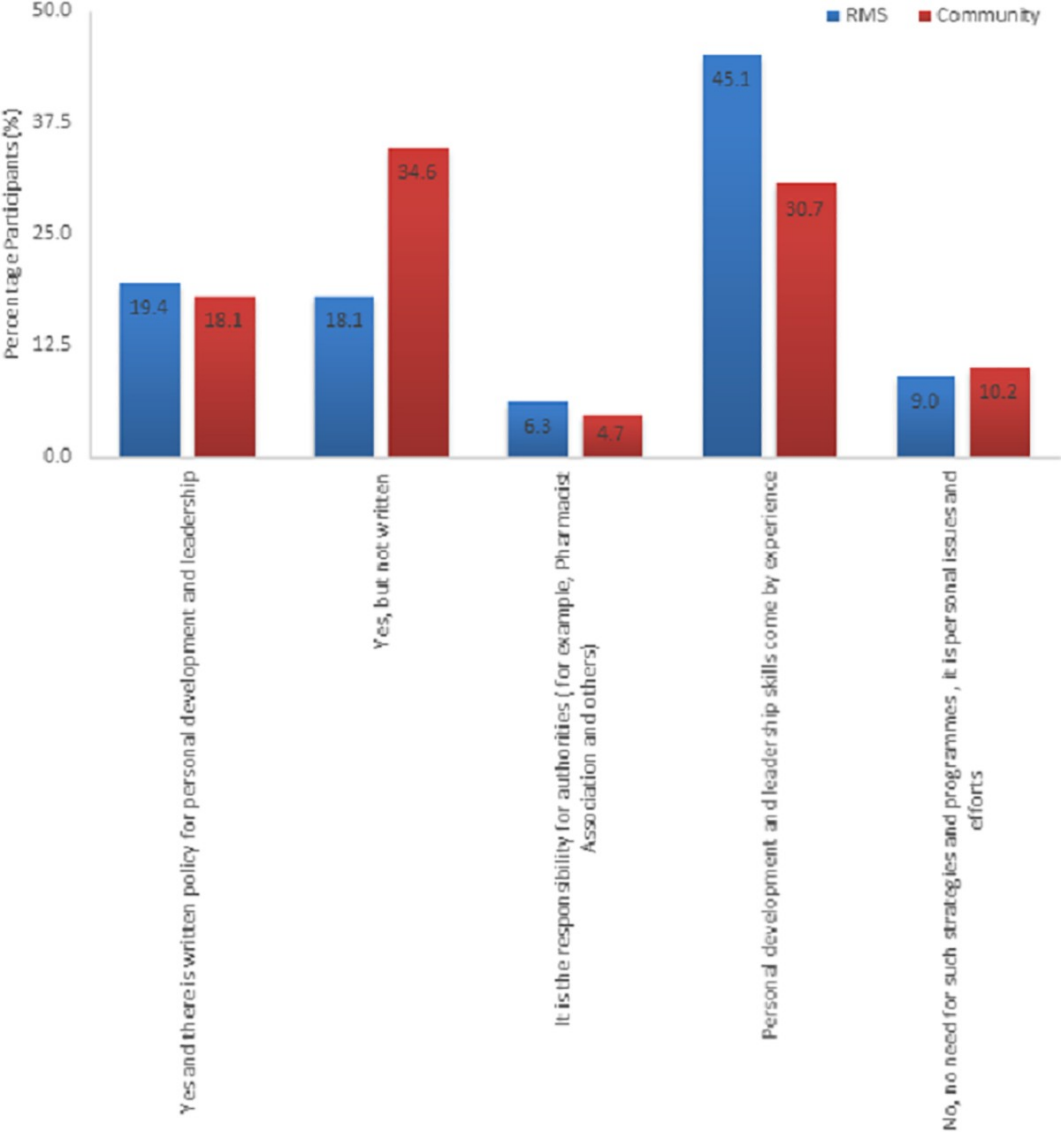
The availability of strategies and programs for personal development and leadership skills in Royal Medical Services and Community Pharmacies.

Unfortunately, only 19.4% of RMS pharmacists and 18.1% of CP confirmed the presence of a written policy for personal development and leadership in their workplace (Fig 2). It could be depicted that many RMS pharmacists 45.1% (N = 65) believed that personal development and leadership skills resulted from experience. Almost one third 34.6% (N = 44) of CP confirmed the availability of non-documented strategies and programs for personal development and leadership. A minority of both RMS and community pharmacies thought that strategies and programs for personal development and leadership were the responsibility of authorities (6.3% and 4.7%, respectively). Written strategies and programs for personal development and leadership skills were significantly higher in RMS compared to community pharmacies (X^2^ = 3.766, df = 1, p = 0.052).

Taking into consideration the specialty fields within the RMS group (Table 5) results show that written policy for personal development and leadership were observed at higher extent within the supply related field compared to the clinical related field (64.4% and 39.3%, respectively). In the same context, higher proportion of staff at supply related field (60%, N = 39) considerd experience as main source for development and leadership skills compared to clinical related field (40%, N = 26). About updating strategies and programs for personal development and leadership in RMS, participants responded equally that the strategies were updated. There was no significant difference in the written strategies for personal development among clinical and supply related fields in the RMS (X^2^ = 0.029, df = 1, p = 0.865).

**Table 5:** The availability of strategies and programs for personal development and leadership based on the specialty field within RMS group.

	Clinical Related				Supply Related			
Strategies and Programs for Personal Development and Leadership	Count	Standard Error of Count	Column N %	Standard Error of Column N %	Count	Standard Error of Count	Column N %	Standard Error of Column N %
Yes and there is written policy for personal development and leadership	11	3	39.3%	9.2%	38	5	64.40%	6.20%
Yes, but not written	10	3	38.5%	9.5%	16	4	61.5%	9.5%
It is the responsibility for authorities (for example, Pharmacist Association and others)	5	2	55.6%	16.6%	4	2	44.4%	16.6%
Personal development and leadership skills come by experience	26	5	40.0%	6.1%	39	5	60.0%	6.1%
No, no need for such strategies and programs, it is personal issues and efforts	6	2	46.2%	13.8%	5	2	38.5%	13.5%

### Incentives and rewards

**Fig 3:**
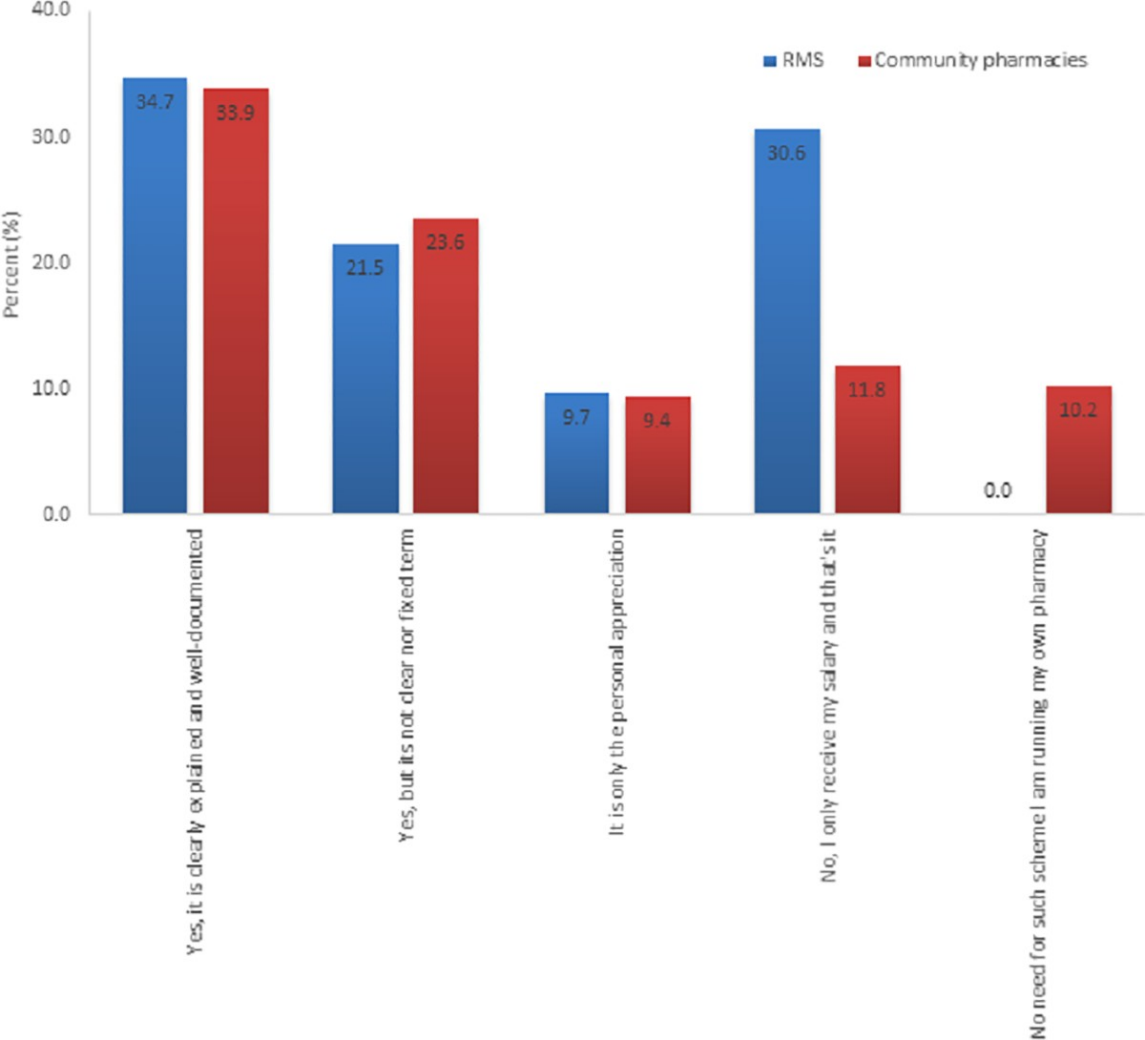
The presence of reward or intensive scheme for good working and superior achievements in Royal Medical Services and Community Pharmacies.

Results show that 34.7% (N = 50) and 33.9% (N = 43) were receiving a clear and well-documented incentive schemes in RMS and CP, respectively. On the other hand, 21.5% (N = 31) and 23.6% (N = 30) were receiving non-fixed scheme of incentives in RMS and CP, respectively. 30.6% (N = 44) of the RMS pharmacists were only receiving their salaries despite of the good working and superior achievements compared to 11.8% (N = 15) for CP. Amazingly, 0% of pharmacists at community pharmacy confirmed that an incentive scheme was unnecessary compared with 10.2% of the RMS staff (Fig 3).

Taking into consideration the specialty fields within the RMS group (Table 6), results show that both clinical and supply related fields are receiving an explained and well documented incentive for research at the same extent. Interestingly, higher proportion of the supply related field pharmacists (57.7%, N = 41) are only receiving their salaries despite their research work compared to (38.0%, N = 27) for the clinical related field pharmacists of the same category. There was no difference in the updating of research incentives between clinical and supply related fields in the RMS (X^2^ = 0.322, df = 1, p = 0.570).

**Table 6:** Relation between specialty fields and research incentives, and whether they are regularly updated in RMS.

		Clinical Related				Supply Related			
Research incentives and updates		Count	Standard Error of Count	Column N %	Standard Error of Column N %	Count	Standard Error of Count	Column N %	Standard Error of Column N %
Incentives	Yes, it is clearly explained and well-documented	21	4	36.2%	6.3%	21	4	25.3%	4.8%
	Yes, but its not clear nor fixed term	10	3	17.2%	5.0%	16	4	19.3%	4.3%
	No, I only receive my salary and that is it	27	5	46.6%	6.5%	41	5	49.4%	5.5%
Regular updating	Yes	18	4	31.0%	6.1%	17	4	20.5%	4.4%
	No	11	3	19.0%	5.1%	14	4	16.9%	4.1%
	I do not know	12	3	20.7%	5.3%	23	4	27.7%	4.9%

### Availability of software programs that aid working practices

**Fig 4:**
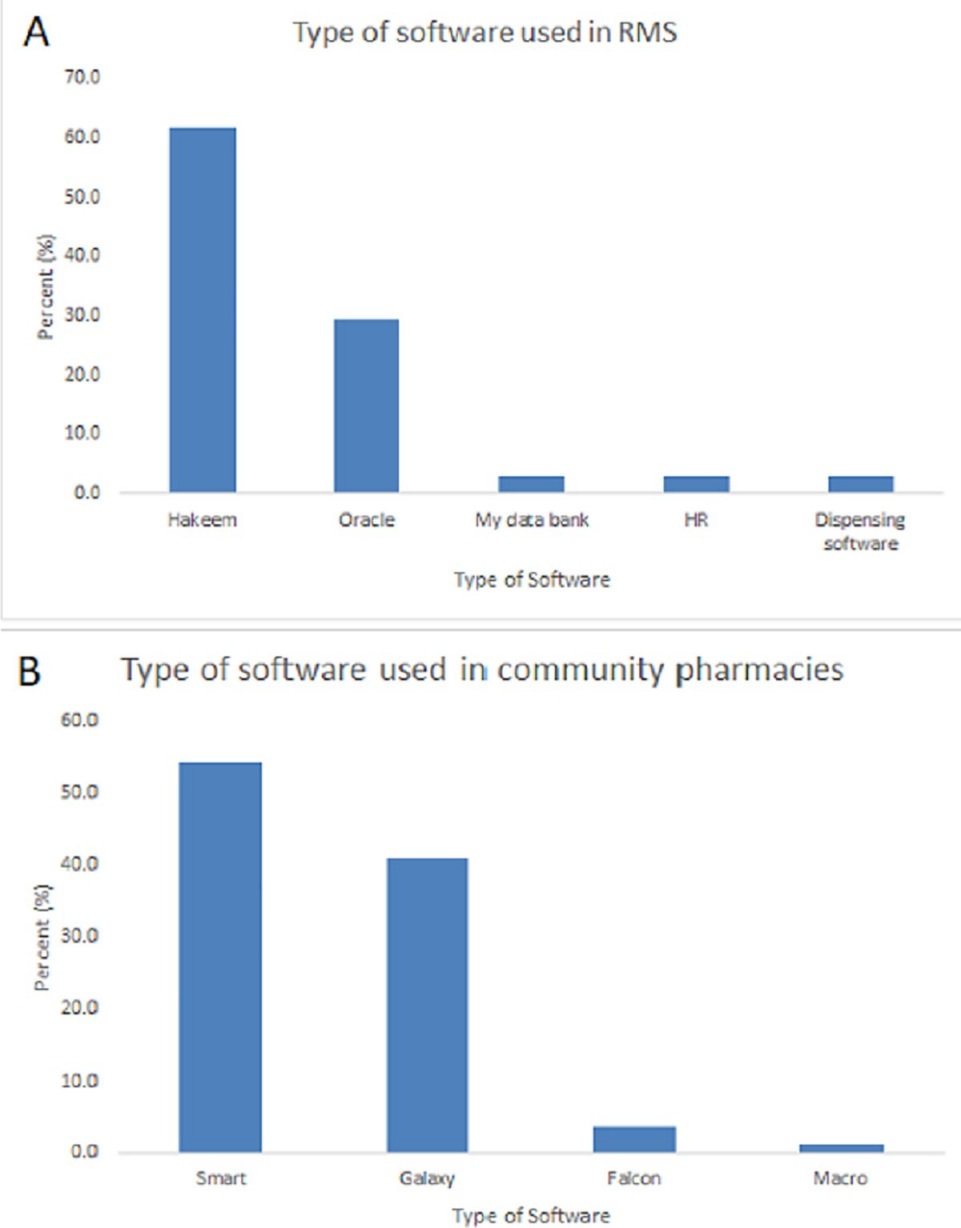
Type of software used in pharmaceutical services. (A) software used in Royal Medical Services (RMS). (B) software used in Community Pharmacies.

The availability of software program that includes all the commercially available medicines along with the available strengths, uses, side effects, drug-drug interactions, prices, and the inventory helps in managing the work. Results show 58.3% (N = 84) and 64.6% (N = 82) have a software program for work management in RMS and CP, respectively. Whereas 38.2% (N = 55) and 29.1% (N = 37) responded that they do not have such programs in the RMS and CP, respectively. The difference in the presence of software at the workplace between RMS and CP was not significant (X^2^ = 1.725, df = 1, p = 0.189). Moreover, no significant difference was found in the presence of software at the workplace between clinical and supply related fields in the RMS (X^2^ = 0.424, df = 1, p = 0.515). Hakeem software was the most used software in RMS (61.8%, N = 42) compared to Smart software in CP (54.2%, N = 45) (Fig 4).

### Actions taken for unknown medical information

**Fig 5:**
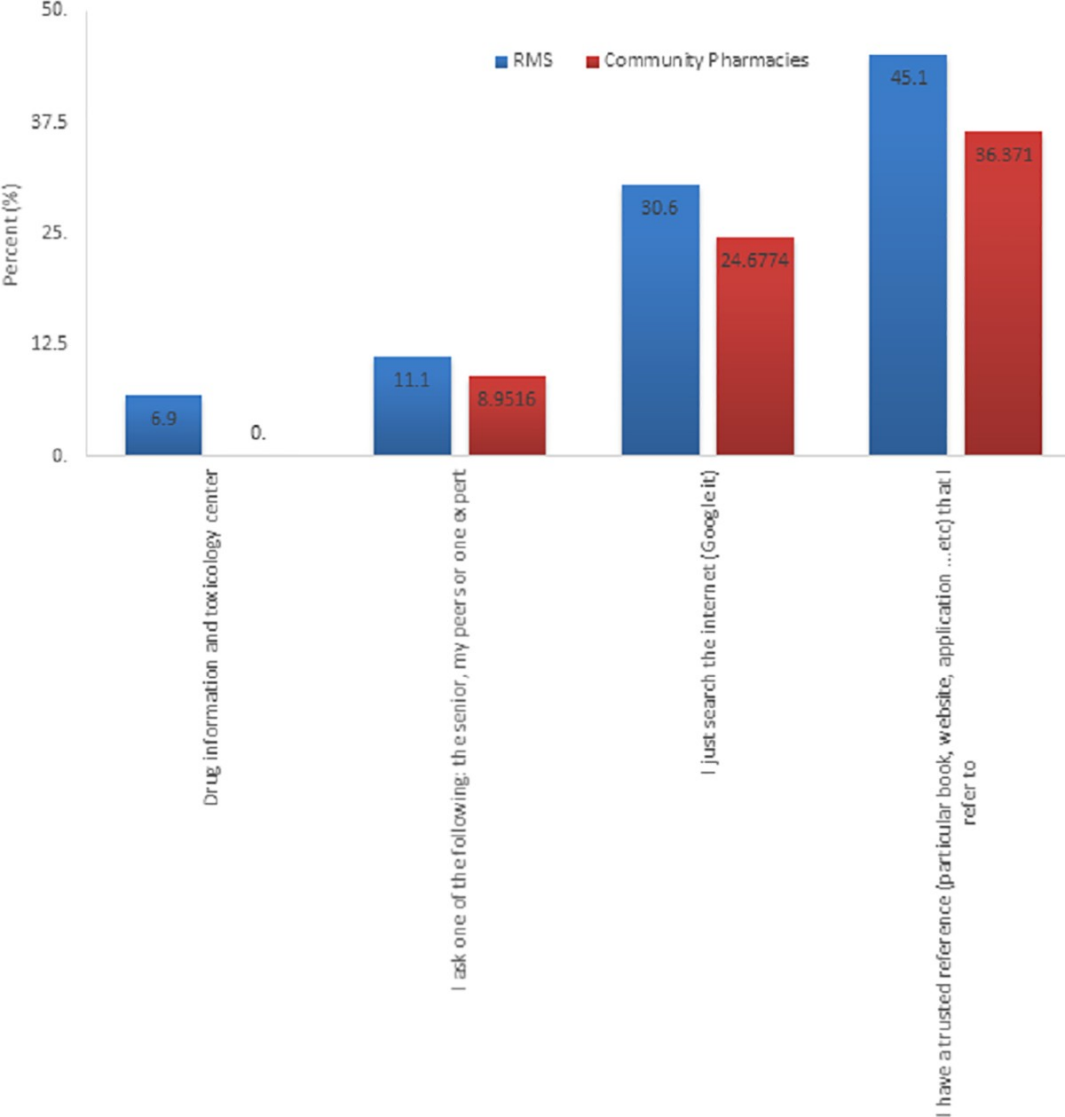
Actions taken for unknown medical information in Royal Medical Services and Community Pharmacies.

Participants were asked what action they take if they require further information to assist them in their duties. The data shows that drug information and toxicology center were not effectively used by CP nor RMS pharmacist. Both groups prefered published references such as books or reputable websites (Fig 5).

### Courses required for enhancing professional development

In a scale from 0 to 10, both groups surprisingly rated the need for having a particular course in the same order as follows (Tables 7 and 8): First, advanced counseling and communication skills (Mean rates for RMS and CP were 8.99±0.145, CI 95% = 3.70–4.28 and 9.37±0.096, CI 95% = 4.18–4.56). Second: Personal development skills (8.92±0.142, CI 95% = 3.64–4.20 and 9.02±0.145, CI 95% = 3.73–4.30). Third, Pharmaceutical health promotion (8.05±0.180, CI 95% = 2.70–3.41 and 8.57±0.159, CI 95% = 3.26–3.89).

**Table 7:** RMS Pharmacist views on courses.

Course Name	N	Mean rate of the perceived need	SE of mean	95% Confidence Interval of the Difference	
				Lower	Upper
**Project Management**	131	7.93*	0.175	2.58	3.28
**Cosmetics**	133	6.45*	0.248	0.96	1.94
**Finance & Economy**	133	7.80*	0.172	2.46	3.14
**Personal Development Skills**	**133**	8.92*	0.142	3.64	4.20
**Pharmaceutical Health Promotion**	**133**	8.05*	0.180	2.70	3.41
**Advanced counselling and Communication Skills**	**133**	8.99*	0.145	3.70	4.28
**Ethical Codes for Medical Practice**	133	7.93*	0.175	3.24	3.84

* P<0.005, based on test value = 5.

**Table 8:** Community pharmacists views on courses that should be required for pharmacists to complete.

Course Name	N	Mean rate of the perceived need	SE of mean	95% Confidence Interval of the Difference	
				Lower	Upper
**Project Management**	127	8.06*	0.173	2.71	3.40
**Cosmetics**	127	8.11*	0.173	2.77	3.45
**Finance & Economy**	127	8.04*	0.187	2.67	3.41
**Personal Development Skills**	**125**	9.02*	0.145	3.73	4.30
**Pharmaceutical Marketing (Promotion)**	**127**	8.57*	0.159	3.26	3.89
**Advanced counselling and Communication Skills**	**127**	9.37*	0.0960	4.18	4.56
**Ethical Codes for Medical Practice**	127	8.2*	0.168	2.87	3.54

* P<0.005, based on test value = 5.

### Traditional modules related to work

Participants were asked about which traditional modules were related to their work (Table 9). For both groups’, participants considered therapeutics as the most commonly used module after graduation (34.3%) followed by Pharmacology (26.6%) The “Over the counter course” was used mostly by CP (37.0%), but not RMS pharmacists (1.4%).

**Table 9:** Traditional modules which mostly were used after graduation.

Course	Community Pharmacists	The Royal Medical Services	Total
Over the counter	47 (37.0%)	2 (1.4%)	49 (18.1%)
Therapeutics	38 (29.9%)	55 (38.2%)	93 (34.3%)
Pharmacology	37 (29.1%)	35 (24.3%)	72 (26.6%)
Pharmaceutical industry	0 (0%)	5 (3.5%)	5 (1.8%)
Physical Pharmacy	1 (0.8%)	0 (0%)	1 (0.4%)
Other	4 (3.1%)	47 (32.6%)	51 (18.8%)
Total	127 (100%)	144 (100%)	271 (100%)

## Discussion

As detailed, the profession of pharmacy is changing in terms of job role- increasing clinical activity, this is accompanied by ever-increasing production and clinical introduction of novel chemical entities and therapeutic technologies [24].

In 2013, the World Health Organization (WHO) issued guidance emphasizing the need for Continuous Education and training for healthcare providers, including pharmacists [45]. The groundwork of pharmacy services is usually based on two factors; robust and well-reputed academic pharmacy institutions and appropriate pharmacist training. The former depends on the academic institution of choice for undergraduate and graduate studies. The latter, however, is a continual process that depends on the stakeholders’ efforts and concern of developing their employees’ knowledge and skills. Requirements for healthcare professional training must have input from all key stakeholders including future employers, existing healthcare professionals, patients and their representatives, local authorities, the professional regulator and government. Collectively they must ensure the sustainable development of an education programs to generate healthcare professionals fit for purpose [46]. Hence, governmental bodies in Jordan which regulate health pharmacutical services such as the Ministry of Health (MOH) and Jordan Pharmaceutical Association (JPA) should set standards, processes, and policies for life-time pharmacists’ education and training to meet current and future needs [47].

The present study outlines the current situation regarding pharmaceutical education and training at both private and public sectors in Jordan, represented by community pharmacies and RMS, respectively. This meets an undocumented area within the field of Jordanian pharmacy practice and education literature. In terms of findings relating to CE and CPD, similar results reporting the lack of structured continuing pharmaceutical education were obtained to those of others [4, 48, 49]. The need for a documented and a well-based CE strategy was raised by Anderson and colleagues [50]. This study emphasized the need for a tailored and documented educational strategy that meets the needs of both the workforces as well as the communities that they serve.

Implementation of leadership training in pharmacy practice is a philosophy that must be considered by pharmacy educators and workforces worldwide. To achieve an implementation of a sustainable leadership system, various elements are required such as administrative and financial support, supportive institutional cultures, focused undergraduate leadership and other ‘soft skills’ courses and activities, and postgraduate and employees’ education and training opportunities [51]. Leadership and self-development skills are typically expected behaviors rather than rewarded skills, therefore individuals with inherited leadership mentality are more willing to actively demonstrate these skills at work. It is observed from the present **study that** 45.1% and 30.7% of RMS and community pharmacists, respectively believe that leadership and development skills come from experience. This echoes the findings of Smith and colleagues [52] who found that pharmacy residences exposed to leadership experience either, validated their prepossessed future interest in a leadership role, or increased their interest in leadership via the obtained skills from non-directed experiences. Such experiences helped participants to obtain leadership skills including decision-making, negotiation, and communication with co-workers.

Our study showed that -in Jordan- there is a need for directed and formal leadership training, only 19.4% and 18.1% of the RMS and CP reported the presence of a written policy for personal development and leadership in their workplace, and many believe that responsible authorities such as the MOH and JPA should provide such training to pharmacists. Zilz and colleagues [53] argued that learning personal development and leadership is a critical component of enabling successful leadership. This entails reading relevant journals and textbooks regularly, as well as involvement in local and international formal programs to boost the required knowledge. The leadership programs should include learning, analyzing, questioning, and mentoring leaders. As detailed in the introduction how best to implement and assess this using validated measures is still an area of debate [1].

The concept of performance incentives for healthcare staff is not well established in developing countries. Within the context of pharmacy there is also the potential for pharmacy businesses to seek financial profits over providing performance-driven reimbursement or incentives [54]. However, recent research and recommendations found that the implementation of pharmacy incentives can enhance pharmacy services [55, 56]. Within our survey participants reported relatively low work incentives in both community and RMS settings. Similar results were obtained in other studies conducted in low and middle-income countries including Iran, Malaysia, Thailand, Bangladesh, Nepal, Pakistan, Yemen Arab Republic, and Syria [54, 57, 58].

Other ways to improve or facilitate CE and CPD is pharmacist engagement with research. Peterson and colleagues report that pharmacists are willing to participate in research to enhance their profession, provide better services, update their knowledge toward disease management, and for personal interest [37]. Pharmacists’ involvement in research not only benefits pharmacists but aids in solving practice-related issues and identification of areas for further research to enhance patient care [36]. However, various challenges face pharmacists’ involvement in research including lack of time, never being approached, low collaborations between academia and pharmacies, and limited finances including grants to facilitate research implementation and buy pharmacists’ time for participation, particularly for community pharmacies [35, 37, 59].

Typically, hospital pharmacies are approached to participate more in research than community pharmacies. This study reflects similar trends where greater engagement with research was noticed in RMS participants compared to CP. In the context of this study, participants were not asked to explain why this might be the case but the authors propose that this difference may be attributed to the economic stress, limited staff numbers profit-making/ sales-oriented base of community pharmacy businesses. However there are numerous examples of successful community based interventions including community pharmacy health checks [60], implementation of extended clinical services through patient group directives [61], COPD screening [62] and pharmacogenomic testing [63] among others.

Reports indicate that financial incentives may be a driving factor for the involvement of pharmacists in research [34, 64]. Nonetheless, pharmacist’s involvement in research will develop their ability to provide evidence-based and improved patients services. Documented and well-structured strategies to increase research engagement include promotions, establishing links between practicing pharmacists and academia, and having access to grants to fund projects are essential for both community and hospital pharmacies [34, 37].

Globally a range of software systems are used to facilitate safe medication management including efficient medicines procurement, decreasing prescription dispensing errors, identification of and avoiding potential drug-related interactions standardization of processes and availability of automated information [65]. The medication system management in both inpatient and outpatient healthcare is a multifactorial interaction system that requires the use of health information technology. Such employment was reported by participants in the present study, where both groups highly reliant on software. Similar results were obtained by other studies [66–69].

## Conclusion

This research shed the lights on professional development perspectives of pharmacists, who working in Royal Medical Services and Community Pharmacies. Development aspects investigated were as follows: continuous education, training infrastructure, leadership skills, rewards and Incentives, using of digital services and management software, finally, exploring options for better academic support delivered to pharmacists.

Taken together, the findings from this study support the crucial need to create a bespoke, written and well documented postgraduate pharmacists’ educational strategies, that encounters not only academic perspectives, but also consider aspects as personal development, and leadership skills. Despite the positive impact of incentive schemes and rewards for superior achievements, such concepts are not effectively utilized to enhance pharmacist’s performance. The present study highlighted many skills that would enhance professional development. The highest rated skills were: Advanced counselling and Communication skills, Personal development skills and pharmaceutical health promotion.

### Recommendations and future work

Based on the received responses, the authors recommend setting guidelines in terms of occupational education and training, personal development and leadership skills training, in addition to schemes of incentives and rewards by the relevant authorities in Jordan such as the Ministry of Health and the Jordan Pharmaceutical Association.

The current work can be extended by expanding the sample size to include other pharmacy-related occupations such as pharmacists working in public and private hospitals, research and development departments in the pharmaceutical factories, pharmacists working in the academic fields, and medical representatives and pharmaceutical marketing teams.

## Supporting information

S1 Data The pdf copy of the survey. (PDF)

S1 Dataset The dataset for responses. (XLSX)
