# Supplementary material for: Postgraduate pharmacist development- an evaluation of Jordanian pharmacist experiences to inform and shape an evidence-based professional development policy
Source: PLoS One. 2021 Jul 27;16(7):e0255026. doi: 10.1371/journal.pone.0255026 (PMC8315534; doi:10.1371/journal.pone.0255026)
Supplement: S1 Data — (PDF) [file pone.0255026.s001.pdf]

# Pharmacists at Royal Medical services

Questionnaire to bridge academic efforts and International Pharmaceutical Federation guidelines to the practical fields
* Information obtained from this survey will be managed according to International Ethical Guidelines, which was prepared by the Council for International Organizations of Medical Sciences (CIOMS), in collaboration with the World Health Organization.
*The information you provide will be published as a report. Confidentiality and anonymity will be maintained and it will not be possible to identify individuals from any publications.
*Your participation is voluntary. You may choose not to participate. If you decide to participate in this research survey, you may withdraw at any time.
*Thank you for your help to enhance the Health care practice.

1. مكان العمل أو القسم

_______________________________

2. Speciality field

*Mark only one oval.*

- ( ) Supply related
- ( ) Clinical related

3. Speciality level

*Mark only one oval.*

- ( ) under training
- ( ) Resident
- ( ) Qulify resident
- ( ) Specialist assisstance
- ( ) Specialist
- ( ) Senior specialist
- ( ) Consultant

## Demographic data

4. Gender

*Mark only one oval.*

- Male
- Female

5. Age group

*Mark only one oval.*

- 22- 30
- 31 - 40
- 41 - 50
- 51 - 60
- 61 - 65
- More than 65

6. From which university you got your degree?

_______________________________

## Professional development

Focus on the pharmaceutical workforce field development

7. At your work place: Is there Pharmaceutical education and training infrastructures?

*Mark only one oval.*

- Yes and there is written policy for training and education
- Yes, but not written
- No, no need for such infrastructure every thing is clear
- It is the responsibility for authorities ( for example, Pharmacist Association and others)
- Education and training comes by experience

8. If you answered above question with yes, is such infrastructure being updated and reviewed on well-known regular basis

*Mark only one oval.*

- Yes
- No
- I do not know

9. At your work place: Is there strategies and programmes for personal development and leadership skills?

*Mark only one oval.*

- Yes and there is written policy for personal development and leadership
- Yes, but not written
- No, no need for such strategies and programmes , it is personal issues and efforts
- It is the responsibility for authorities ( for example, Pharmacist Association and others)
- Personal development and leadership skills come by experience

10. If you answered above question with yes, are such programmes being updated and reviewed on well-known regular basis

*Mark only one oval.*

- Yes
- No

11. At your work place: Is there any means of reward or incentive scheme for good working and superior achievements?

*Mark only one oval.*

- Yes, it is clearly explained and well-documented
- Yes, but its not clear nor fixed term
- It is only the personal appreciation
- No need for such scheme I am ruining my own pharmacy
- No, I only receive my salary and that it is
- Other answer

12. If you answered above question with yes, are such incentive being updated and reviewed on well-known regular basis

*Mark only one oval.*

- Yes
- No
- I do not know

13. At your work place: Is there any means of reward or incentive scheme for research based work?

*Mark only one oval.*

- Yes, it is clearly explained and well-documented
- Yes, but its not clear nor fixed term
- No, I only receive my salary and that it is

14. If you answered above question with yes, are such incentive being updated and reviewed on well-known regular basis

*Mark only one oval.*

- Yes
- No
- I do not know

15. At your work place: is there a software program that helps managing the work ?

*Mark only one oval.*

- Yes, there is a program and I use it
- Yes, there is a program but I do not use it frequently
- No, we do not have such program

16. If there is a software program, what is it?

17. if you have a software program what would you suggest to make it more useful for your work

18. In your work: If you do not know some thing related to medicines, what is your first action

*Mark only one oval.*

- I have a trusted reference (particular book, website, application ...etc) that I refer to
- I just search the internet (Google it)
- I ask one of the following: the senior, my peers or one expert
- Drug informationa and toxicology centre
- Other

19. Generally, what is the best on line electronic references you recommend others to use for drug's related information? if more than one please list them in order

20. Do you have a direct and full access to the reference(s) you recommended in the question above?

*Mark only one oval.*

- Yes
- No

## Academic development and related issue

21. Which of the following fields are most related to your work in pharmacy ?

*Mark only one oval.*

- Pharmacology
- Therapeutics
- Medicinal Chemistry
- Pharmaceutical industry
- Physical Pharmacy
- Pharmacognosy
- OTC
- Other

22. To which extent you agree that the following course (Project Management) is needed for Pharmacists?

*Mark only one oval.*

| 1 | 2 | 3 | 4 | 5 | 6 | 7 | 8 | 9 | 10 |
|---|---|---|---|---|---|---|---|---|---|
| | | | | | | | | | |

23. To which extent you agree that the following course (Cosmetics) is needed for Pharmacists?

*Mark only one oval.*

| 1 | 2 | 3 | 4 | 5 | 6 | 7 | 8 | 9 | 10 |
|---|---|---|---|---|---|---|---|---|---|
| ⬭ | ⬭ | ⬭ | ⬭ | ⬭ | ⬭ | ⬭ | ⬭ | ⬭ | ⬭ |

24. To which extent you agree that the following course (Finance and Economy) is needed for Pharmacists?

*Mark only one oval.*

| 1 | 2 | 3 | 4 | 5 | 6 | 7 | 8 | 9 | 10 |
|---|---|---|---|---|---|---|---|---|---|
| ⬭ | ⬭ | ⬭ | ⬭ | ⬭ | ⬭ | ⬭ | ⬭ | ⬭ | ⬭ |

25. To which extent you agree that the following course (Personal Development skills) is needed for Pharmacists?

*Mark only one oval.*

| 1 | 2 | 3 | 4 | 5 | 6 | 7 | 8 | 9 | 10 |
|---|---|---|---|---|---|---|---|---|---|
| ⬭ | ⬭ | ⬭ | ⬭ | ⬭ | ⬭ | ⬭ | ⬭ | ⬭ | ⬭ |

26. To which extent you agree that the following course (Pharmaceutical Health Promotion) is needed for Pharmacists?

*Mark only one oval.*

| 1 | 2 | 3 | 4 | 5 | 6 | 7 | 8 | 9 | 10 |
|---|---|---|---|---|---|---|---|---|---|
| ⬭ | ⬭ | ⬭ | ⬭ | ⬭ | ⬭ | ⬭ | ⬭ | ⬭ | ⬭ |

27. To which extent you agree that the following course (Advance counselling and communication skills) is needed for Pharmacists?

*Mark only one oval.*

| 1 | 2 | 3 | 4 | 5 | 6 | 7 | 8 | 9 | 10 |
|---|---|---|---|---|---|---|---|---|---|
| ◯ | ◯ | ◯ | ◯ | ◯ | ◯ | ◯ | ◯ | ◯ | ◯ |

28. To which extent you agree that the following course (Ethical codes for medical practice) is needed for Pharmacists?

*Mark only one oval.*

| 1 | 2 | 3 | 4 | 5 | 6 | 7 | 8 | 9 | 10 |
|---|---|---|---|---|---|---|---|---|---|
| ◯ | ◯ | ◯ | ◯ | ◯ | ◯ | ◯ | ◯ | ◯ | ◯ |

29. What other course(s) would you recommend to be added to the pharmacy curriculum? If more than one kindly list them according to priority

30. You may would like to explain and support your recommendation in the last question

31. Workshops: in the following questions If a university would like to support you with a building capacity training, which workshop you would attend? scale from 1-5 (Sponsored by the university, free for participants)

_______________________________

32. Professional coaching skills

*Mark only one oval.*

| 1 | 2 | 3 | 4 | 5 |
|---|---|---|---|---|
| ◯ | ◯ | ◯ | ◯ | ◯ |

33. Professional time managment

*Mark only one oval.*

| 1 | 2 | 3 | 4 | 5 |
|---|---|---|---|---|
| ◯ | ◯ | ◯ | ◯ | ◯ |

34. Personal communication skills

*Mark only one oval.*

| 1 | 2 | 3 | 4 | 5 |
|---|---|---|---|---|
| ◯ | ◯ | ◯ | ◯ | ◯ |

35. Team work (building teams and drive success)

*Mark only one oval.*

| 1 | 2 | 3 | 4 | 5 |
|---|---|---|---|---|
| ⬭ | ⬭ | ⬭ | ⬭ | ⬭ |

36. Leadership related topics

*Mark only one oval.*

| 1 | 2 | 3 | 4 | 5 |
|---|---|---|---|---|
| ⬭ | ⬭ | ⬭ | ⬭ | ⬭ |

37. If non of the suggested workshops could attract you, Please suggest other workshops

38. If you would have further official education, which of the following options you would choose ?

*Mark only one oval.*

- Professional Diploma in Pharmaceutical Management
- Professional Diploma in Pharmaceutical Marketing
- Professional Diploma in Research Methods
- Professional Diploma in Human Resource Management
- Professional Diploma in Leadership
- Others, Master degree
- Other, PhD degree
- Others

39. In your opinion: How can Academic institutions (universities) support your development?

This content is neither created nor endorsed by Google.

Google Forms
